# Microencapsulation of lemongrass and mangosteen peel as phytogenic compounds to gas kinetics, fermentation, degradability, methane production, and microbial population using *in vitro* gas technique

**DOI:** 10.1371/journal.pone.0304282

**Published:** 2024-06-05

**Authors:** Rittikeard Prachumchai, Chaichana Suriyapha, Gamonmas Dagaew, Sukruthai Sommai, Maharach Matra, Srisan Phupaboon, Yupin Phasuk, Metha Wanapat

**Affiliations:** 1 Department of Animal Science, Faculty of Agricultural Technology, Rajamangala, University of Technology Thanyaburi, Pathum Thani, Thailand; 2 Tropical Feed Resources Research and Development Center (TROFREC), Department of Animal Science, Faculty of Agriculture, Khon Kaen University, Khon Kaen, Thailand; Zagazig University Faculty of Agriculture, EGYPT

## Abstract

The purpose of the current study was to evaluate the impact of various doses of microencapsulated lemongrass and mangosteen peel (MELM) on gas dynamics, rumen fermentation, degradability, methane production, and microbial population in *in vitro* gas experiments. With five levels of microencapsulated-phytonutrient supplementation at 0, 1, 2, 3, and 4% of substrate, 0.5 g of roughage, and a concentrate ratio of 60:40, the trial was set up as a completely randomized design. Under investigation, the amount of final asymptotic gas volume was corresponding responded to completely digested substrate (*b*) increased cubically as a result of the addition of MELM (*P* < 0.01) and a cubic rise in cumulative gas output. The amount of MELM form did not change the pH and NH_3_-N concentration of the rumen after 12 and 24 h of incubation. However, methane production during 24 h of incubation, the levels were cubically decreased with further doses of MELM (*P* < 0.01) at 12 h of incubation. Increasing the dosage of MELM supplementation at 2% DM resulted in a significant increase in the digestibility of *in vitro* neutral detergent fiber (IVNDF) and *in vitro* true digestibility (IVTD) at various incubation times (*P* < 0.05), but decreased above 3% DM supplementations. Moreover, the concentration of propionic acid (C3) exhibited the variations across the different levels of MELM (*P* < 0.05), with the maximum concentration obtained at 2% DM. The populations of *Fibrobacter succinogenes*, *Ruminococcus albus*, *Ruminococcus flavefaciens*, and *Megasphaera elsdenii* revealed a significant increase (*P* < 0.05), while the quantity of Methanobacteriales decreased linearly with increasing doses of MELM. In conclusion, the inclusion of MELM at a concentration of 2% DM in the substrate which could enhance cumulative gas production, NDF and true digestibility, C3 production, and microbial population, while reducing methane concentration and Methanobacterial abundance.

## Introduction

The production of methane (CH_4_) from ruminants is a significant contributor to global warming, accounting for approximately 18% of total CH_4_ emissions worldwide [1]. This issue not only affects the environment but also results in energy loss (8%-12%) and reduced feed efficiency [2, 3]. In order to address this problem, one tactic is to manipulate the rumen in order to improve rumen performance, maximize feed consumption, and reduce environmental pollution [4]. One such strategy that has demonstrated potential for increasing rumen fermentation and lowering greenhouse gas emissions is feeding ruminants fruit peels that contain phytochemical substances [5]. Previous research suggests that adding plant species high in phytonutrient compounds to animal diets, like raw banana [6], dragon fruit [5], rambutan (*Nephelium lappaceum* L.) [7], and royal poinciana seed meal (*Delonix regia*) [8], can raise levels of ruminal propionic acid (C3) and microbial protein synthesis while lowering protozoal populations and rumen CH_4_ gas production. As a result, there is a need for the exploitation of plant products used as feed additives to increase feed efficiency in ruminant nutrition and production, as well as to attenuate rumen CH_4_, which has been increasingly of interest and concern [4].

Total polyphenolic content (TPC) and total flavonoid content (TFC), by-products of tropical agriculture found in mangosteen peel, have the potential to fight off gram-positive microbes by preventing the synthesis of cell walls and cytoplasmic membranes [2, 3]. The utilization of mangosteen (*Garcinia mangostana* L.) peel as a phytonutrient source in ruminant diets has the potential to assist in the reduction of CH_4_ and biohydrogenation, while simultaneously maintaining optimal ruminal pH and volatile fatty acids (VFA) levels and decreasing the methanogenic population inside the rumen [9]. This might prove beneficial for beef cattle and swamp buffaloes [6, 10]. Moreover, another plant that is of interest is lemongrass (*Cymbopogun citratus*), which may enhance animal output. In the tropics and subtropics, lemongrass is a common native plant that is full of bioactive substances like flavonoids, phenols, essential oils and high citral content required for vitamin A synthesis. According to certain investigations, lemongrass has advantages for animal production, ruminal fermentation, fatty acid profile, antioxidant activity, and total bacterial population while having no adverse effect on ruminant health [11]. Nevertheless, the depletion of bioactive substances from phytonutrients may have an impact on the chemical composition’s stability as well as its vulnerability to certain microorganisms [12]. Several approaches have been developed to enhance the quantity of a nutrient that passes through the rumen without being degraded by rumen microbes, resulting in the delivery of a larger part of that nutrient to the lower gastrointestinal tract [13]. The microencapsulation technology is widely used in the agriculture, feed, and pharmaceutical sectors. This technology is also suitable to the ruminant feed business, since it shields nutrients from degradation in the rumen, allowing for increased bioavailability of the main element in the small intestine [14].

Our hypothesis was that the microencapsulation of bioactive components from lemongrass and mangosteen peel would alter the ruminal microbiota to improve gas dynamics and ruminal fermentation, reducing methane emissions. The objective of the present study was to investigate the impact of various doses of a microencapsulated blend of phytonutrients on the microbial population, utilizing diets supplemented with MELM, with respect to gas dynamics, rumen fermentation, degradability, and methane production on *in vitro* study.

## Materials and methods

### Animal ethics

The approval protocol no. IACUC-KKU-110/66 was issued by the Animal Welfare Committees of Khon Kaen University.

### Bioactive molecule extraction, preparation, and microencapsulation

To extract bioactive components from lemongrass and mangosteen peel, two procedural approaches were applied: microwave extraction (MIE) and maceration extraction (MAE) [13]. For the MIE method, in order to achieve a final temperature of 60°C, microwave power of 100 W was applied to 30 g of powdered sample and 300 mL of deionized water for 35 minutes. The sample was agitated with a methanol solution at room temperature overnight for the MAE technique. In order to prepare the encapsulated formulation later, the supernatant was then filtered through cellulose filter paper, collected in a vacuum glass bottle, and kept at 4°C.

With the use of an EnSight multimode plate reader (PerkinElmer Inc., Waltham, MA, USA), the TPC, TFC, and antioxidant capacity of the bioactive component extracts were assessed. The process adhered to the guidelines provided by Phupaboon et al. [14]. TFC was assessed using colorimetric changes with 10% aluminum chloride solution at 415 nm [15], whilst TPC was calculated by measuring absorbance at 765 nm using the Folin-Ciocalteu reagent [16]. The findings of the analyses were represented as mg GAE/g DM and mg QUE/g DM, and they were performed in triplicate.

Following the procedures described by Nouri [17] and Kurek and Pratap-Singh [18], the bioactive solution extracted from lemongrass and mangosteen peel was microencapsulated using a technique involving ionic gelation mixed with surfactant components. As described in the current research, minor alterations were made. A 1% (v/v) acetic acid solution was used to dissolve the chitosan, which was then mixed with a 2% (v/v) concentration of Tween 80 surfactant. The liquid was steadily mixed until it reached a homogeneous consistency at a temperature of 65°C. The wall material was mixed with the bioactive extract juice in a 1:1 (v/v) ratio and swirled continuously at room temperature for the length of an overnight period to accomplish encapsulation. The microencapsulated mixtures were spray dried (Buchi Mini spray dryer, Laguna Hills, CA, USA). The material was dried using a peristaltic pump, which transported it into the drying chamber at a rate of 10 mL/min and 110 L/h of airflow with a pressure drop of 0.73 bar. While the input temperature was 160°C, the output temperature was kept at 90°C. The resulting dry powders were gathered, hermetically sealed, and stored at 20°C before being used in an *in vitro* experiment.

### Treatment, animal’s management and rumen fluid inoculum

The study was designed as a completely randomized design (CRD) with five levels of microencapsulated lemongrass and mangosteen peel (MELM) 0, 1, 2, 3, and 4% DM with 0.5 g of roughage and concentrate ratio of 60:40. The chemical composition ratios of the concentrates, MELM, and rice straw are shown in Table 1.

**Table 1:** Ingredient and chemical composition of concentrate diet, rice straw, and microencapsulated of lemongrass and mangosteen peel (MELM) (%DM).

Item	Concentrate	MELM	Rice Straw
Ingredients, (% DM)			
Cassava chips	43.72		
Corn meal	16.26		
Rice bran	11.03		
Soybean meal (SBM)	10.88		
Palm kernel meal	15.17		
Minerals and vitamins^1^	0.91		
Urea	1.11		
Salt	0.92		
lemongrass		50.00	
mangosteen peel		45.00	
Cassava starch		5.00	
Chemical composition			
Dry matter, %	93.94	83.17	93.70
Organic matter, %DM	94.61	88.58	88.96
Crude protein, %DM	15.04	18.66	2.13
Neutral detergent fiber, %DM	20.12	42.62	68.71
Acid detergent fiber, %DM	12.81	1.92	42.62
Bioactive Compounds			
TPC (mg GAE/g DM)		125.42	
TFC (mg QUE/g DM)		219.7	

^1^Minerals and vitamins (per kg): Vitamin A = 10,000,000 IU, Vitamin E = 70,000 IU, Vitamin D = 1,600,000 IU, Fe = 50 g, Zn = 40 g, Mn = 40 g, Co = 0.1 g, Cu = 10 g, Se = 0.1 g, I: 0.5 g.

TPC = total polyphenolic content; TFC = total flavonoid content

MELM = microencapsulated of lemongrass and mangosteen peel

Four Thai native steers weighing 350 ± 10 kg and aged 2.0 to 2.3 years were used to supply the necessary rumen fluid. Animals were offered a concentrate composed of 14% DM crude protein (CP), 22% DM neutral detergent fiber (NDF), 11% DM acid detergent fiber (ADF), and total digestible nutrient of 75.6% DM. The steers received rice straw twice daily at 7:30 AM and 3:30 PM for a total of 14 days, or 0.5% of their body weight. Each individual steer was housed in its own cage and had full access to mineral blocks and clean water. Prior to their morning feed intake, the pump connected to the stomach tube was performed through the mouth to collect rumen fluids from each steer. An equal volume of rumen fluid from four steers was pooled and transferred into warmed thermos flasks with an oxygen-free headspace. This fluid was then brought to the lab at 39°C in anaerobic conditions. The liquids were filtered through four layers of cheesecloth before being utilized as an inoculum.

### In vitro rumen fermentation and gas determination

The protocols outlined by Poungchompu et al. [10] were adhered to in the *in vitro* analysis and the production of the artificial saliva and rumen fluid. The following rumen medium preparations were mixed with 660 mL of rumen fluid in an anaerobic environment: distilled water (1095 mL), a trace mineral mixture (0.23 mL), a macro mineral mixture (365 mL), a resazurin mixture (1 mL), a reduction mixture (60 mL), and a buffer mixture (730 mL). Prior to incubation, a portable pH meter was employed to determine whether the rumen fluid is within the normal pH range of 6.5–7.0. The 0.5 g samples of rice straw and concentrate were very carefully placed within 50 ml serum vials. The *in vitro* gas generation test was carried out by incubating the vials for 96 h at a temperature of 39°C after being completely closed with rubber stoppers and crimp caps. Every three hours, the vials were occasionally stirred during the incubation. As baseline benchmarks for each measurement period, the research also included five vials containing solely rumen fluid and their average gas production values. The bottles were divided into four sets: the first set (4 bottles × 5 treatments + 4 bottles of blank) was used for gas kinetics and gas production measurement; the second set (4 bottles × 5 treatments × 2 observation times at 12 and 24 h + 4 bottles of blank) was used for measurement of ruminal parameters including pH, ammonia-nitrogen (NH_3_-N), VFA, microbial community, and CH_4_. For the analysis of VFA and NH_3_-N, a total of 9 ml of liquid samples were employed. Furthermore, the 44 bottles were prepared separately for microbial community studies (4 bottles × 5 treatments × 2 observation times at 12 and 24 h + 4 bottles of blank). The third set (4 bottles × 5 treatments × 2 observation times at 12 and 24 h + 4 bottles of blank) was used for determination of DM and OM degradability; and the last set (4 bottles × 5 treatments × 2 observation times at 12 and 24 h + 4 bottles of blank) was used for determination of NDF degradability.

### Chemical analysis and measurements

The diet, MELM, and rice straw samples were passed through a 1-mm sieve (Cyclotech Mill, Tecator, Hoganas, Sweden). The samples were firstly ground and then dried at 60°C for 72 h. After drying, *in vitro* examination was performed on the samples to ascertain their chemical concentration and gas kinetics. Dry matter (DM) (ID 967.03), organic matter (OM) (ID 942.05), and acid detergent fiber (ADF) measurements were made in accordance with the AOAC International Method [19] and residual ash was also determined and taken into account. According to the methods described by Van Soest et al. [20], neutral detergent fiber (NDF) was measured using α-amylase and sodium sulfite (Sigma no. A3306, Sigma Chemical Co., St. Louis, MO). Following the instructions published by the AOAC [19], the crude protein (CP) (ID 984.13) content of the feed samples was analyzed using a Leco combustion nitrogen analyzer (Leco CN628 Carbon/Nitrogen Analyzer, Leco Instruments Inc., St. Joseph, MI).

A total 96 h of a time series of incubation at 0.5, 1, 2, 4, 6, 8, 12, 18, 24, 36, 48, 72, and 96 h. were performed to measure the gas production using a pressure transducer syringe. Total gas = *b* × [1 − exp^-c(t-L)^] was used to calculate the cumulative gas production curve. *b* is the final gas volume produced after complete substrate digestion (in mL/g DM), t is the incubation period (in hours), c is a rate constant (in time units), and L is a discontinuous lag term (in hours) Schofield [21].

At 12 and 24 h after incubation, 20 samples from 40 bottles containing fermented inoculum were taken for pH measurements (Hanna instrument HI 8424 microcomputer, Pte. Ltd., m 161 Kallang Way, Singapore). Then, Grade 40 Cheesecloth was used to filter the liquid. The ruminal fluid samples were centrifuged at 16,000 × g for 10 minutes at 4°C, and the supernatant and rumen digesta was stored in the freezer at –20°C. Thawed ruminal and digesta samples were then tested for VFA, NH3-N, and microbial community. The technique described by So et al. [22] was modified by Yamamoto et al. [23] to estimate the fraction of VFA using gas chromatography. Centrifuged rumen fluid (0.8 ml) is combined with 1.6 ml of diethyl ether, 0.3 ml of 50% H_2_SO_4_, and 0.8 ml of 5 mmol/L 2-methylvaleric acid (Kanto Chemical). A 15-minute centrifugation at 2500 rpm was then applied to the mixture. Afterwards, a test tube holding around 0.1 g was filled with 1 ml of ether layer, and the test tube was let to stand for 5 minutes. The ether extract was then put into a vial tube and injected into a gas chromatography unit (Model HP6890, Hewlett Packard, Rockville, MD, USA) using a front injector (syringe size 10 μl, injection volume 1 μl), valve (box heater at 150°C), inlet (heater 200°C, pressure 20.45 psi, total flow 12 ml/min, septum purge flow 3 ml/min, mode as split at 5:1 ratio with split flow 7.5 ml/min), oven (oven temp at 120°C, initial at 115°C and ramp 1 at 120°C), and detector (heater 200°C, air flow 400 ml/min, H2 fuel flow 40 ml/min, makeup flow of N2 25 ml/min, column flow of He 1.5 ml/min). NH_3_-N was analysed according to the method of Fawcett and Scott [24] approach, a 15 ml test tube equipped with a vortex mixer was employed to amalgamate 40 microliters of centrifuged rumen liquid, 2500 microliters of phenol color reagent, and 2000 microliters of alkaline hypochloride reagent. A blue reaction took place after vortexing and ten minutes at 37°C in an agitating water bath. The combination was then subjected to a second examination at 630 nm with a UV/Vis spectrophotometer. After each gas pressure measurement, a subsample of gas (2.5 mL) was collected into 10 mL vacuum tubes to determine the methane (CH_4_) concentration using a gas chromatograph (Shimadzu GC 2014, Chiyodaku, Tokyo, Japan) equipped with flame ionization detector (FID) and a capillary HP-molesieve column (GC 30 m × 0.53 mm × 25 μm). The GC conditions were a column temperature of 60°C, injector temperature of 200 °C, detector temperature of 240 °C, and the carrier gas (Helium) in constant flux at 10 mL/min. A calibration curve was prepared using 99.5% pure CH_4_ standard (Praxair Industrial Gases, Osasco, Brazil) [25].

In order to evaluate the substance’s *in vitro* dry matter digestibility (IVDMD), it was filtered through glass filter crucibles that had been pre-weighed and then incubated for 12 and 24 h (with four samples for each treatment) [26]. To determine the quantity of OM and the digestibility percentage of OM (IVOMD), the glass filter crucibles were subjected to a heating process at a temperature of 550°C for a duration of 6 h. The results were based on the residual data. The *in vitro* NDF degradability (IVNDFD) at 12 and 24 h of incubation was performed by pouring the samples from each bottle into Ankom bags (pre-weighed and recorded the weight) and emptying the bottles with distilled water, then the bags were brought to oven drying at 100 C for 24 hours and weighed. After that, the bags were closed and subsequently put into the analysis process of NDF using Ankom fiber analyzer to estimate the IVNDFD [20]. The NDF concentration of the indigestible residues in the test bottles was determined in order to compute the *in vitro* true digestibility (IVTD) using the following equations.

IVTD=100−[(100−NDFD)×(NDF/100)],

where NDF = neutral detergent fiber (% of DM), IVTD = *in vitro* true digestibility (% of DM), and NDFD = neutral detergent fiber digestibility (% of NDF).

### Microbial community in the rumen

At 12 and 24 h after incubation, 0.5-mL aliquots of digesta and rumen fluid were used to extract community DNA using the RBB+C technique, as outlined by Yu and Morrison [27]. To put it briefly, 500 mM NaCl, 50 m M EDTA, and 4% (w/v) sodium dodecyl sulfate (SDS) are added to the mixture while bead-beating the cells to lyse them. Additionally, as DNases are very active in the rumen and gastrointestinal sample, the buffer ought to shield the released DNA from being broken down by them. Following bead-beating, the majority of contaminants and SDS are eliminated by precipitating ammonium acetate, and the nucleic acids are then eliminated by precipitating isopropanol. Following sequential digestion with RNase A and proteinase K, genomic DNA may be purified. Columns from the QIAGEN DNA Mini Stool Kit (QIAGEN, Valencia, CA, USA) are used to collect the DNA. *Fibrobacter succinogenes* was amplified under standard PCR conditions. These conditions included 30 seconds of denaturation at a temperature of 94°C, 30 seconds of annealing at 60°C, and 30 seconds of extension at 72°C. There were a total of 48 rounds of this procedure. The last cycle’s extension time was 10 minutes, compared to the first cycle’s denaturation duration of 9 minutes. Amplification of 16S rRNA for *Ruminococcus flavefaciens* and *Ruminococcus albus* was defined in a similar method, with the exception that the annealing temperature was set to 55°C according to Kolike and Kobayashi [28]. The extracted genomic DNA, SYBR Green PCR Master Mix, forward and reverse primers, and template DNA from Applied Biosystems in Warrington, UK, were used in the real-time quantitative PCR tests. The populations of *F*. *succinogenes*, *R*. *flavefaciens*, and *R*. *albus* [28], *Butyrivibrio fibrisolvens* and *Megasphaera elsdenii* [29], and methanogenic archaea [30] were all quantified using specific primers [31]. The Chromo 4TM system (Bio-Rad, CA, USA) for real-time PCR amplification and detection was used to quantify DNA standards.

### Statistical analysis

The *in vitro* trial data were analyzed with PROC GLM from SAS [32] using a completely randomized design. The model used is displayed as follows:

$$Y_{ij}=\mu +M_{i}+\varepsilon_{ij}$$

In this instance, Yij stands for the response variables, while μ denotes the overall mean. With i ranging from 0 to 4% of the substrate, M denotes the influence of different dosages of MELM. The symbol εij denotes the residuals. The responses’ means were supplied in addition to the mean standard error. The statistical analysis used the least significant difference (lsd) technique, with a significance threshold set at *P* < 0.05, to evaluate differences between treatment means. Orthogonal polynomial contrasts (linear, quadratic, and cubic) were used to assess the impact of MELM supplementation.

## Results

### Kinetics of gas and total gas production

**Fig 1:**
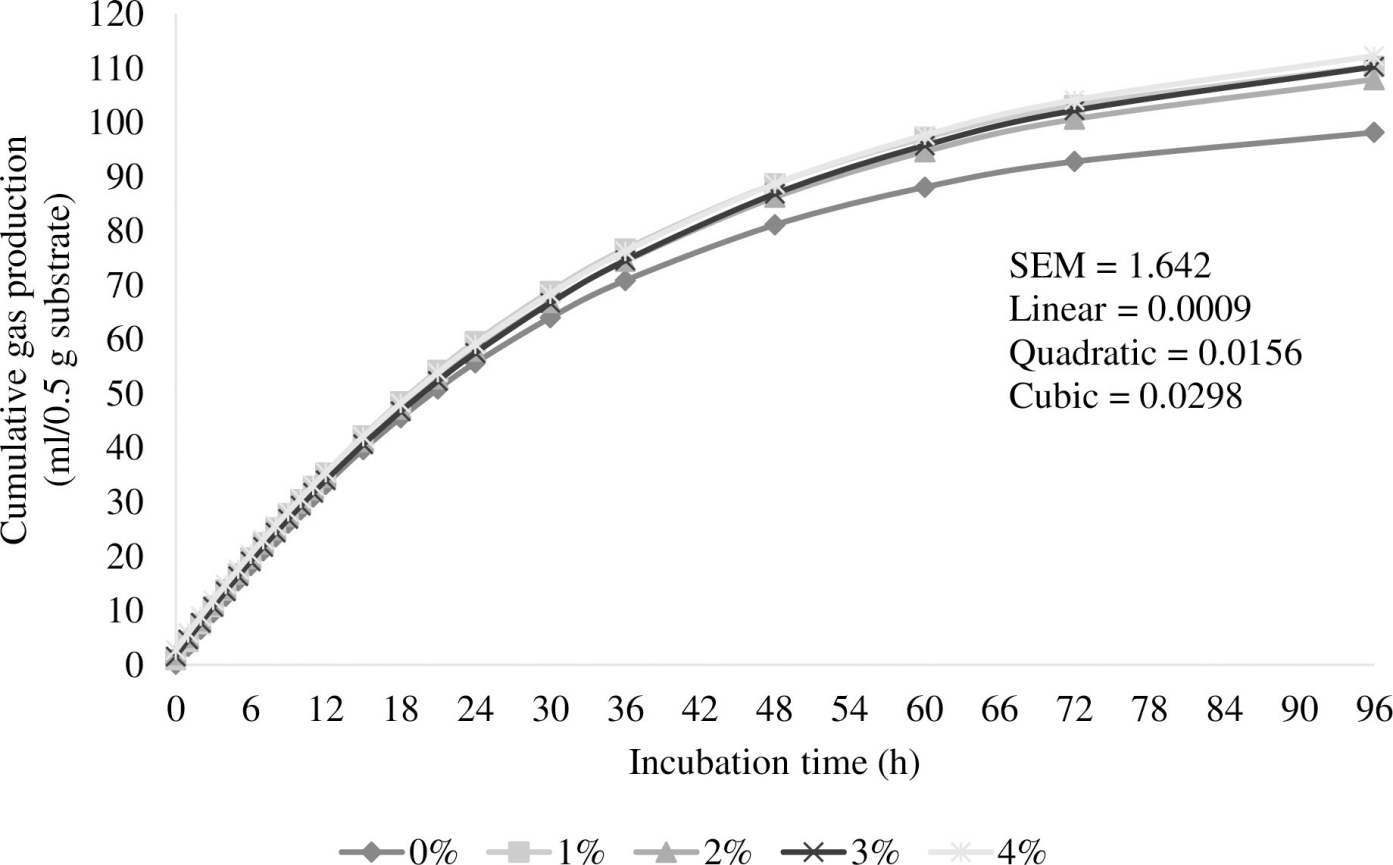
Effect of cumulative gas during incubation durations of microencapsulated lemongrass and mangosteen peel (MELM) levels. The total amount of gas produced or accumulated during a given time when the samples were held under controlled circumstances for 0 to 96 hours.

Fig 1 and S1 Fig exhibit the information pertinent to the effect of microencapsulated of lemongrass and mangosteen peel (MELM) levels on cumulative gas during incubation times, and the kinetics of the gas production parameters as well as the cumulative gas production observed at 96 h of incubation are listed in Table 2. Gas production rate (c) and discrete lag time (L) did not differ significantly (*P* > 0.05). The ultimate asymptotic gas volume corresponding to completely digested substrate (b) increased cubically as a result of the addition of MELM (*P* < 0.01) and cumulative gas generation. Increases in the MELM level beyond 1% in 0.5 g substrate had an impact on the highest values of kinetic gas "*b*" and cumulative gas production throughout 96 h of incubation, which were 121.25 mL/0.5 g and 112.28 mL/g DM, respectively.

**Table 2:** Effect of microencapsulated of lemongrass and mangosteen peel (MELM) level on gas kinetics and cumulative gas at 96 h after incubation.

Level of MELM (%)	Gas kinetics (mL/0.5 g DM)			Cumulative Gas (mL/g DM Basis)
	*b*	c	L	
0	102.77^b^	0.12	0.40	98.17^b^
1	117.48^a^	0.03	0.28	110.36^a^
2	115.47^a^	0.03	0.23	107.95^a^
3	119.13^a^	0.03	0.20	110.28^a^
4	121.25^a^	0.03	0.01	112.28^a^
SEM	1.826	0.030	0.116	1.642
Contrasts				
Linear	0.0001	0.0698	0.2710	0.0009
Quadratic	0.0150	0.1810	0.7193	0.0156
Cubic	0.0248	0.5335	0.8952	0.0298

^a-b^Means in the same column with different lowercase letters differ (*P* < 0.05); SEM = standard error of the mean; *b* = the final asymptotic gas volume corresponding to fully digested substrate (mL/g DM); c = a rate constant (units time 1); L = a discontinuous lag term (h); MELM = microencapsulated of lemongrass and mangosteen peel

### Ruminal pH, NH_3_-N concentration, CH_4_ production, and volatile fatty acid (VFA) concentration

The effects of MELM on *in vitro* ruminal pH, NH_3_-N concentration, CH_4_ production, and volatile fatty acid (VFA) concentration are shown in Table 3. In 12 and 24 h incubation, the amount of MELM did not affect the pH of the rumen (*P* > 0.05), with range values of 6.84 to 6.95 and 6.84 to 6.88, respectively. Different dosages of MELM supplementation had no effect on the NH_3_-N concentration (*P* > 0.05), which varied between 12.48 to 16.64 and 18.31 to 21.84 mg/dL after 12 and 24 h of incubation, respectively. The addition of MELM had no effect on methane production at 24 h of incubation (*P* > 0.05), but in 12 h incubation, the levels decreased cubically with increasing dosages (*P* < 0.01). Moreover, Different dosages of MELM supplementation had no appreciable impact on the content of TVFA, C2, or C4 (*P* > 0.05). C3 concentration varied across different levels of MELM (*P* < 0.05), with the highest concentration found at 2% DM.

**Table 3:** Effect of microencapsulated of lemongrass and mangosteen peel (MELM) level on ruminal pH, ammonia-nitrogen (NH_3_-N) concentration, methane production, and volatile fatty acid (VFA) concentrations.

Item	Level of MELM (%)					SEM	Contrasts		
	0	1	2	3	4		Linear	Quadratic	Cubic
Rumen parameters									
pH									
12 h	6.95	6.92	6.92	6.94	6.92	0.013	0.8296	0.0764	0.9143
24 h	6.88	6.88	6.85	6.84	6.88	0.019	0.1073	0.6171	0.5045
NH_3_-N (mg/dL)									
12 h	12.48	13.00	13.39	14.29	16.64	0.834	0.1615	0.8351	0.8839
24 h	19.26	18.31	18.40	20.05	21.84	1.347	0.6963	0.3850	0.9395
Methane (mL/1 g dry matter substrate)									
12 h	4.86^a^	2.25^c^	3.52^b^	2.40^c^	2.01^c^	0.247	0.0063	0.0569	0.0057
24 h	6.75	6.98	5.32	5.79	6.11	0.888	0.4804	0.9261	0.4670
Total VFA (mmol/l)									
12 h	61.39	64.22	61.25	60.19	62.74	1.352	0.3253	0.2094	0.2593
24 h	70.08	73.47	73.32	77.16	77.11	3.899	0.3962	0.9654	0.7477
Acetate (C2) (%)									
12 h	70.04	70.30	71.48	70.03	71.01	0.461	0.5916	0.0935	0.1179
24 h	67.07	65.86	65.87	67.13	67.32	0.469	0.9345	0.0784	0.9940
Propionate (C3) (%)									
12 h	17.49	17.38	16.59	17.74	17.10	0.483	0.9813	0.0609	0.0666
24 h	19.67^b^^c^	21.11^a^^b^	21.23^a^	20.13^a^^b^^c^	19.19^c^	0.353	0.4376	0.0253	0.9667
Butyrate (C4) (%)									
12 h	12.47	12.31	11.94	12.23	11.89	0.287	0.6720	0.9453	0.0958
24 h	13.26	13.03	12.89	12.74	13.49	0.545	0.5644	0.9538	0.9765

^a-c^Means in the same column with different lowercase letters differ (*P* < 0.05); SEM = standard error of the mean; MELM = microencapsulated of lemongrass and mangosteen peel

### In vitro digestibility

The effect of MELM on nutritional *in vitro* digestibility is shown in Table 4. At 12 h of incubation, no significant changes (*P* > 0.05) were seen in the digestibility of dry matter (DM) or organic matter (OM). Nevertheless, the addition of MELM supplementation had quadratic effects on the digestibility of DM and OM in 24 h incubation (*P* < 0.01). The addition of MELM at 4% DM in 0.5 g substrate resulted in a considerable decrease in digestibility. The lowest recorded values for DM digestibility were 48.70% and 55.10% for OM digestion. Furthermore, increasing the dose of MELM supplementation at 2% DM resulted in a substantial improvement in IVNDF and IVTD digestibility at various incubation durations (*P* < 0.05), but decreased above 3% DM supplementation.

**Table 4:** Effect of microencapsulated of lemongrass and mangosteen peel (MELM) level on *in vitro* digestibility of nutrients.

Item	Level of MELM (%)					SEM	Contrasts		
	0	1	2	3	4		Linear	Quadratic	Cubic
*In vitro* dry matter digestibility (IVDMD) (% DM)									
12 h	42.68	42.40	43.24	41.91	40.02	0.733	0.6953	0.5149	0.3458
24 h	51.98^a^^b^	53.79^a^	53.94^a^	51.12^b^	48.70^c^	0.643	0.4213	0.0049	0.6598
*In vitro* organic matter digestibility (IVOMD) (% DM)									
12 h	49.70	50.67	51.37	49.90	48.00	0.804	0.7513	0.1872	0.6141
24 h	60.00^a^^b^	61.47^a^	60.35^a^	58.70^b^	55.10^c^	0.437	0.0351	0.0099	0.3714
*In vitro* NDF digestibility (IVNDFD) (% DM)									
12 h	54.00^b^	54.40^a^^b^	56.01^a^	53.25^b^	52.60^b^	0.561	0.8053	0.0204	0.0528
24 h	59.61^a^^b^	59.77^a^^b^	60.74^a^	58.09^b^^c^	57.52^c^	0.572	0.1932	0.0360	0.1163
*In vitro* true digestibility (IVTD) (% DM)									
12 h	77.34^b^	77.53^a^^b^	78.33^a^	76.97^b^	76.64^b^	0.276	0.8083	0.0202	0.0529
24 h	80.10^a^^b^	80.18^a^^b^	80.66^a^	79.35^b^^c^	79.07^c^	0.282	0.1921	0.0364	0.1167

^a-c^Means in the same column with different lowercase letters differ (*P* < 0.05); SEM = standard error of the mean; MELM = microencapsulated of lemongrass and mangosteen peel

### Microbial population

Table 5, significant effects of level of MELM were observed for all microoganism except *B*. *fibrisolvens*. The populations of *F*. *succinogenes*, *R*. *albus*, *R*. *flavefaciens*, and *M*. *elsdenii* exhibited a significant increase (*P* < 0.05) when the dosage of MELM was elevated. The maximum population levels were observed when the substrate was supplemented with 2% DM. In contrast, the quantity of Methanobacteriales at 12 h of incubation reduced linearly with raising doses of MELM, with the lowest value being 2.19 × 10^9^ copies/mL for MELM supplementation at 4% DM (*P* < 0.01).

**Table 5:** Effect of microencapsulated of lemongrass and mangosteen peel (MELM) level on microbial abundance.

Item	Level of MELM (%)					SEM	Contrasts		
	0	1	2	3	4		Linear	Quadratic	Cubic
Copies/mL of rumen content,									
*F*.*succinogenes*, ×10^9^									
12 h	0.32^c^	1.18^b^	2.28^a^	0.42^c^	0.90^bc^	0.179	0.1439	0.0006	0.0102
24 h	2.32^b^	3.55^a^	3.74^a^	2.09^b^	1.46^b^	0.217	0.6836	0.0041	0.4602
*R*. *albus*, ×10^10^									
12 h	0.79^b^	1.58^ab^	2.09^a^	1.29^ab^	1.29^ab^	0.162	0.1088	0.0279	0.3328
24 h	2.83^b^	2.51^b^	3.94^a^	2.67^b^	2.15^b^	0.233	0.4173	0.1090	0.0130
*R*. *flavefaciens*, ×10^9^									
12 h	2.46^c^	3.12^c^	6.00^a^	4.36^b^	2.33^c^	0.280	0.0048	0.0214	0.0064
24 h	3.65^ab^	3.78^ab^	5.04^a^	3.03^b^	2.49^b^	0.346	0.7190	0.0369	0.0464
*M*. *elsdenii*, ×10^9^									
12 h	5.38^c^	7.58^bc^	15.13^a^	8.33^b^	7.55^bc^	0.417	0.0078	0.0031	0.0021
24 h	10.00^c^	13.62^b^	22.90^a^	10.52^c^	13.59^b^	0.410	0.0007	0.0004	0.0159
*B*. *fibrisolvens*, ×10^8^									
12 h	5.49	4.44	4.75	4.24	5.75	0.321	0.8511	0.9444	0.8899
24 h	4.58	3.70	5.35	5.59	3.36	0.123	0.4319	0.6665	0.5025
Methanobacteriales, ×10^9^									
12 h	5.72^a^	4.50^ab^	3.40^bc^	3.25^bc^	2.19^c^	0.409	0.0056	0.2495	0.6701
24 h	2.80	2.05	2.52	1.90	1.69	0.439	0.3055	0.8856	0.2916

^a-c^Means in the same column with different lowercase letters differ (*P* < 0.05); SEM = standard error of the mean; MELM = microencapsulated of lemongrass and mangosteen peel

## Discussions

MELM at concentrations greater than 1% of the substrate tends to increase the ultimate asymptotic gas volume associated with full substrate digestion (*b*) and total gas generation. This may be due to the inclusion of lemongrass and mangosteen peel, two plants high in flavonoids and polyphenolic compounds. The fermentable organic degradability during substrate fermentation was enhanced, which significantly increased the overall gas output. This finding is congruent with the findings of Purba et al. [33], who discovered that adding 15–45 mg of Piper betle powder to an *in vitro* gas study increased total gas production, especially when combined with a low-quality roughage source like rice straw. Similar results were found in a research by Kim et al. [34], which demonstrated that after 72 h of incubation, the total gas production in the presence of all flavonoid-rich plant extracts was considerably higher than that of the control group.

Ruminal pH was unaffected by the addition of lemongrass and mangosteen peel amounts. The measured values of pH complied with the optimum reference range for microbial function given by Ørskov and Ryle [35]. The study conducted by Wanapat et al. [9] provided evidence that the inclusion of lemongrass in the diet did not exert any significant impact on the pH levels of rumen fluid. The study conducted by Polyorach et al. [36] revealed that the administration of mangosteen peel powder to lactating dairy cows at several doses of 100, 200, and 400 g/head/day did not result in significant alterations in ruminal pH. In addition, ruminal NH_3_-N was unaffected by higher quantities of MELM. According to Preston and Leng [37] and Wanapat and Pimpa [38] studies, ruminal NH_3_-N levels between 15–30 mg/dL were shown to have a significant beneficial influence on rumen fermentation and to be favorable for microbial activity. These results align with the outcomes of the current investigation, which reported ruminal NH_3_-N values of 12.48–21.48 mg/dL. These results are consistent with those of Ampapon et al. [39], who found that the administration of Chaya powder mixed with rambutan peel, both of which contain a wealth of phytonutrients, had no discernible effect on the level of ruminal NH_3_-N. Nevertheless, the data given in this study contradict the findings of Wanapat et al. [9], who reported that the incorporation of lemongrass or mangosteen into cows’ feed led to a reduction in hyper-ammonia-producing bacteria. Consequently, this resulted in a drop in the rate of ammonia-N generation from amino acids.

At 12 h of incubation, the control group demonstrated a methane production of 4.86 mL/g DM. Methane generation was significantly reduced when MELM were added, with values of 2.25, 3.52, 2.40, and 2.01 mL/g DM, respectively. The breakdown of flavonoids during rumen fermentation is responsible for this occurrence. By having a suppressive impact on the population of methanogenic bacteria, flavonoids have the ability to lower the production of CH_4_. Flavonoids have inhibitory effects on protozoa’s cytoplasmic membrane function and nucleic synthesis, according to Cushnie and Lamb [40]. Researchers Wang et al. [41] and Seradj et al. [42] found that feeding flavonoids to cattle decreased the overall population’s protozoa count. Ma et al. [43] reported that the addition of mulberry leaf meal, enriched with flavonoids, caused a reduction in the protozoa’s population, which in turn decreased the production of CH_4_. Numerous researchers have demonstrated the beneficial effects of plant phytonutrients on methane reduction and the population of methanogens in both *in vitro* and *in vivo* studies [44].

The addition of MELM caused a quadratic change in the values of *in vitro* dry matter digestibility (IVDMD), *in vitro* organic matter digestibility (IVOMD), *in vitro* NDF digestibility, and *in vitro* true digestibility (IVTD). When compared to the control group, increasing the dose of MELM at 2% DM tended to improve the value of digestibilities for DM, OM, NDF, and IVTD. According to Cherdthong et al. [45], the provision of flavonoids and polyphenolic compounds resulted in the supply of crucial nutrients to enhance rumen microbial activity, hence potentially enhancing nutrient digestion and improving *in vitro* digestibility. Nevertheless, the *in vitro* digestibility saw a decline after a 24-hour incubation period when the quantities of MELM were at 4% dry matter in 0.5 g of substrate. According to Cieslak et al. [46], it has been observed that soluble sugar has the potential to interact with phytochemicals, resulting in a reduced physical availability of soluble sugar for rumen microbes. The activity of the microbe is reduced as a result of this interaction, and DM and OM are less digestible. Furthermore, the higher flavonoid content seen in lemongrass and mangosteen may be responsible for the observed behavior. Through their interactions with bacterial enzymes and/or the formation of complexes with indigestible cell wall carbohydrates, these flavonoids have the power to reduce the digestibility of cell walls. In the study conducted by Scalbert [47], it was shown that there are three distinct mechanisms by which phytonutrients might exhibit toxicity towards microbes. These mechanisms include: 1. blockage of enzymes and deprivation of substrates, 2. impact on cellular membranes, and 3. deprivation of essential metal ions.

In this study, the addition of MELM at a concentration of 2% DM resulted in a considerable enhancement in the molar percentage of C3. This discovery maybe relates to the influence of phytonutrients on the bacterial composition within the rumen. The increase in the bacterial population seen in this experimental work has the potential to positively impact rumen fermentation, resulting in an overall rise in the production of VFA and propionate. According to Ampapon and Wanapat [7], the incorporation of rambutan peel powder into the diet of beef cattle at a dry matter intake of 4% led to an increase in rumen propionate production. Furthermore, Khiaosa-Ard and Zebeli [48] conducted a study that showed the injection of phytonutrients had a notable effect on increasing propionate levels and decreasing acetate concentration in dairy cows. In a study conducted by Totakul et al. [44], it was demonstrated that the incorporation of flavonoids, namely in the form of chaya leaf meal pellet, into the dietary regimen of dairy cows resulted in improvements in both VFA and propionate levels.

The study demonstrated that the administration of the MELM supplement resulted in a notable increase in the populations of *F*. *succinogenes*, *R*. *albus*, and *R*. *flavefaciens*. One possible explanation for this occurrence is the presence of phytogenic chemicals in animal feed, which may exist in their original form or as extracts derived from plants. Numerous studies have provided evidence indicating that these chemicals possess the capacity to exert an impact on the mitigation of methane generation within the rumen environment, a process mostly mediated by rumen microbial populations. Moreover, they exert a direct influence on distinct microorganisms, including protozoa and cellulolytic bacteria. Significantly, previous research undertaken by Buccioni et al. [49] and Correa et al. [50] has demonstrated that these chemicals have the ability to augment the populations of *F*. *succinogenes*, *R*. *albus*, *B*. *fibrisolvens*, and *R*. *flavefaciens*. Dairy cows given Samanea saman (rain tree pod meal) containing phytonutrients had an increase in the population of *F*. *succinogenes*, likely as a result of a decrease in the number of protozoa and methanogens, according to Anantasook et al. [51]. Wanapat et al. [52] reported that the inclusion of 100 g of mangosteen peel per head per day in the diet of swamp buffaloes resulted in an augmentation of *R*. *flavefaciens* and other rumen bacteria, accompanied by a reduction in methanogens. The presence of flavonoids in MELM has been found to have advantageous effects on ruminal function, hence contributing to the preservation of performance and overall well-being [53]. This study investigates the impact of flavonoids on rumen fermentation dynamics, specifically focusing on the increase in propionate percentage. Additionally, the study explores the potential of flavonoids to promote the proliferation of beneficial microbes, such as *M*. *elsdenii*, which are known for their ability to use lactate. These findings suggest that the inclusion of flavonoids in animal diets may have favorable implications for animal performance.

While these MELM considerably reduced the number of Methanobacteriales. The decline in methane-producing microorganisms corresponds to the observed reduction in methane output. The activity of rumen microbes can be directly affected by flavonoids [54], or indirectly influenced by new derivatives that are produced during biotransformation or degradation processes [55]. Moreover, Patra et al. [56] observed that extracts containing phenolics exhibited a reduction in ruminal methane emission and methanogenesis count. Dong et al. [57] conducted a study whereby they observed that the incorporation of Moringa oleifera, a plant species abundant in phytonutrients, led to alterations in the composition and diversity of methanogenic microorganisms. Consequently, this intervention demonstrated a significant reduction in methane emissions among dairy cows.

## Conclusions and recommendation

In conclusion, the inclusion of MELM at a concentration of 2% DM in the substrate which could enhance cumulative gas production, NDF and true digestibility, C3 production, and microbial population, while reducing methane concentration and Methanobacterial abundance. Therefore, using MELM could be an option for potential by enhancing the to increase rumen fermentation efficiency and with no negative effects on rumen ecology. However, further investigation through *in vivo* trials is necessary to assess its impact on CH_4_ production decline and methanogens in ruminants.

## Supporting information

S1 Fig The total amount of gas produced by microencapsulated mangosteen peel and lemongrass (MELM) during the 0 to 96 hours of incubation. (XLSX)
